# A new cerebral vessel benchmark dataset (CAPUT) for validation of image-based aneurysm deformation estimation algorithms

**DOI:** 10.1038/s41598-018-34489-2

**Published:** 2018-10-30

**Authors:** Daniel Schetelig, Andreas Frölich, Tobias Knopp, René Werner

**Affiliations:** 1 University Medical Center Hamburg-Eppendorf, Department of Computational Neuroscience, Hamburg, 20246 Germany; 2 University Medical Center Hamburg-Eppendorf, Department of Diagnostic and Interventional Neuroradiology, Hamburg, 20246 Germany; 3 University Medical Center Hamburg-Eppendorf, Section for Biomedical Imaging, Hamburg, 20246 Germany; 4 Hamburg University of Technology, Institute for Biomedical Imaging, Hamburg, 20246 Germany

## Abstract

Hemodynamic properties and deformation of vessel structures are assumed to be correlated to the initiation, development, and rupture of cerebral aneurysms. Therefore, precise quantification of wall motion is essential. However, using standard-of-care imaging data, approaches for patient-specific estimation of pulsatile deformation are prone to uncertainties due to, e.g., contrast agent inflow-related intensity changes and small deformation compared to the image resolution. A ground truth dataset that allows evaluating and finetuning algorithms for deformation estimation is lacking. We designed a flow phantom with deformable structures that resemble cerebral vessels and exhibit physiologically plausible deformation. The deformation was simultaneously recorded using a flat panel CT and a video camera, yielding video data with higher resolution and SNR, which was used to compute ‘ground truth’ structure deformation measures. The dataset was further applied to evaluate registration-based deformation estimation. The results illustrate that registration approaches can be used to estimate deformation with adequate precision. Yet, the accuracy depended on the registration parameters, illustrating the need to evaluate and finetune deformation estimation approaches by ground truth data. To fill the existing gap, the acquired benchmark dataset is provided freely available as the CAPUT (Cerebral Aneurysm PUlsation Testing) dataset, accessible at https://www.github.com/IPMI-ICNS-UKE/CAPUT.

## Introduction

A cerebral aneurysm is an acquired pathological local dilation of the diameter of a vessel wall, which is associated with a structural weakening of the vessel wall^1^. The weakened structural integrity results in a depletion of the wall strength^2^. When the circumferential stresses, due to heart cycle-related pressure variations, surpass the (reduced) wall strength, the risk of rupture increases significantly^3^. Rupture and the following subarachnoid hemorrhage are associated with dangerous complications, ranging from severe cognitive or physical impairments to fatality^4,5^. Despite the extreme consequences of aneurysm rupture, the initiation, growth, and development of aneurysms are still not entirely understood and are probably processes dependent on multiple factors^6^. Decision finding for optimal treatment of aneurysms (e.g. clipping, coiling, monitoring) currently incorporates only aneurysm size and location as controlling factors. Yet, multiple studies suggest that the complex interaction of mechanical and physiological factors (vessel deformation, hemodynamics) plays a considerable role during pathogenesis and ultimately rupture^7–9^. The underlying hypothesis is that aneurysm deformation could be correlated to corresponding wall stresses, which, in turn, could (considering the biomechanics of vessel structures) be linked to a risk of rupture. Respective quantitative data could in principle be valuable information to refine existing guidelines for treatment^9^, but reliability and robustness of existing methods to derive them in standard-of-care image data need to be improved for application in clinical routine.

In previous work, we focussed on the effects of contrast agent inflow on the quantification of wall motion using a non-deforming flow phantom^10^. Our results revealed a dependency of the intensity changes due to contrast agent inflow on the extent of estimated wall deformation, therefore illustrating, that computational methods used in high-precision studies such as the quantification of cerebral wall motion have to be fine-tuned and verified using ground truth data. Our current study extends this work and addresses the uncertainties of image-based analysis of related hemodynamic properties, specifically the deformation of aneurysm structures using a deformable flow phantom. Currently, one essential limitation in the context of automated estimation of pulsatile deformation of aneurysms and wall motion of vessel structures is the lack of ground truth data. Proposed methods for vessel structure deformation estimation (e.g. manual threshold-based methods^11^ and semi-automated registration-based methods^12^) are prone to uncertainty due to, for instance, challenges caused by intensity changes as the result of contrast agent inflow and limited spatial/temporal resolution of the image data. The influence of these challenges affects the accuracy of deformation estimation. However, the accuracy cannot be reliably assessed using only *in vivo* radiology imaging data, which limits the reliability of respective patient-specific vessel wall motion estimation. Furthermore, related deformation quantification uncertainties propagate into the computation of derived measures like associated stresses, rendering them also questionable.

**Figure 1:**
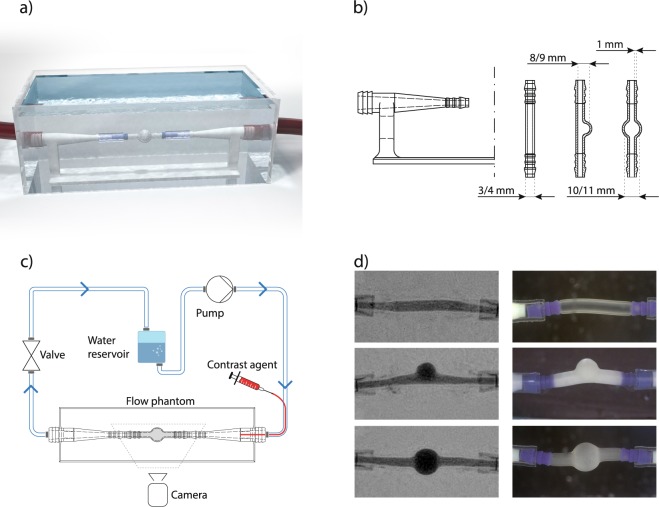
Flow phantom design and schematic representation of experimental setup. (**a**) Computer-aided design, (**b**) technical drawing of the phantom structures (flow distributor, straight tube, saccular aneurysm, fusiform aneurysm), (**c**) experimental setup, (**d**) exemplary FPCT (left) and video (right) data, from top to bottom: straight tube, saccular aneurysm & fusiform aneurysm.

Accordingly, the aim and contribution of the current study are twofold. First, we generated and provide a new benchmark dataset, the so-called CAPUT (Cerebral Aneurysm PUlsation Testing) dataset, that can be used for evaluation of frameworks for cerebral wall motion estimation. Therefore, we constructed a novel deforming flow phantom (see Fig. 1a). The central part of the phantom is an exchangeable 3D-printed deformable structure, which in our case resembles anatomical structures (saccular aneurysm, fusiform aneurysm, artery) and is made out of deformable rubber polymer. During the experiment, cyclic changes in pressure are generated to produce a deformation of the flow phantom structure. The deformation of the flow phantom structures is simultaneously recorded using a flat panel computer tomography (FPCT) and an external video camera, with the high spatial resolution video data providing reliable ground truth for structure deformation evaluation.

As second contribution, we illustrate the application of the benchmark data to evaluate the accuracy of automated wall motion estimation by deformable image registration (DIR). As part of the analysis, we also investigate how precise a deformation can be quantified in medical image sequences with, in comparison to the FPCT data, significantly reduced spatial and temporal resolution as commonly found for other imaging modalities like computed tomography (CT) and magnetic resonance imaging (MRI)^11,13–16^. This addresses the inherent conflict between the low resolution of respective standard-of-care clinical image data and the necessity for high precision regarding the wall motion estimation, and, consequently, the validity of previous findings of studies employing CT and MRI data for cerebral wall motion quantification (for an overview of related studies see^9^). The structure of the paper follows the afore-mentioned contributions. In the section “Flow Phantom and Benchmark Data”, the flow phantom design is explained and the benchmark data introduced. The experiments on the accuracy of DIR-based quantification of wall motion quantification are described in the subsequent section. Respective results are presented and discussed in the “Results” and “Discussion”. The paper is concluded with final remarks.

## Flow Phantom and Benchmark Data

### Flow phantom design

The flow phantom was designed to provide a repeatable, physiologically reasonable deformation of vessel-like structures. The entire measurement setup is displayed in Fig. 1c. It consists of a water circuit, powered by a pump. The pump can be regulated to indirectly adjust the generated pressure and the resulting deformation of the deformable phantom structures. The continuous flow provided by the pump is interrupted using a valve to imitate the mechanical action of the heart. The valve is triggered using a motor driver with an H bridge, controlled by an Arduino-microcontroller board. The interruption of the liquid stream leads to changes in flow velocity and pressure and, in turn, to a deformation of the central structure(s) of the flow phantom (see exemplary video provided as supplemental material). Contrast agent can be directly applied into the central structures using a catheter. The central structures (connected to the flow circuit by inflow/outflow structures, which are designed to reduce the formation of turbulent flow and dampen the mechanical agitation of the whole system by external forces) resemble vascular anatomy (artery, saccular aneurysm, fusiform aneurysm) and are 3D-printed using a deformable material (TangoBlackPlus, shore hardness: A27, tensile strength: 0.8–1.5 MPa, elongation at break: 170–220%). All structures are designed with two inner diameters of the feeding vessels (diameter 1 = 4 mm, diameter 2 = 3 mm, wall thickness = 1 mm) in order to generate deformations of different extent. This leads to aneurysm diameters ranging from 8 to 11 mm at the aneurysm dome (for scenario with 4 mm inner diameter of the feeding vessel structure: fusiform aneurysm inner diameter: 11 mm, saccular aneurysm inner diameter: 9 mm; for scenario with 3 mm inner diameter of the feeding vessel structure: fusiform aneurysm inner diameter: 10 mm, saccular aneurysm inner diameter: 8 mm). Technical drawings of the structures and inflow/outflow structure can be found in Fig. 1b. The entire flow phantom was placed inside a water bath to simulate the soft tissue environment of the vessels (Fig. 1a).

### Benchmark data acquisition and open access

Flat panel computer tomography image data was acquired using a Philips AlluraXper (10 ml contrast agent, Imerson 400, Dilution: 50%). The spatial resolution for the FPCT sequences was 0.185 × 0.185 mm with a temporal resolution of 25 FPS. During the experiments, the valve was triggered with a frequency of 1 Hz, resembling a physiologically plausible heart rate. The pressure of the pump was varied to produce anatomically plausible deformations for the different flow phantom structures. For planning purposes, the deformation was estimated analytically (for calculations see supplemental materials S1). Given the results of previous work (*in vivo* and *in vitro* experiments^9,12,17^), we generated pulsatile deformation of the flow phantom structures in the range of 0.08 to 0.25 mm.

The video data, recorded simultaneously to FPCT data acquisition, was captured with a spatial resolution of 1920 × 1080 px and again using a temporal resolution of 25 FPS. To record the wall motion as precisely as possible, a macro lens (focal length: 105 mm) was used. Since the video files did not contain dedicated pixel spacing information, pixel spacing calibration had to be performed manually. Therefore, the extent of a non-deforming structure of the flow phantom was measured in pixels and compared to its known geometric extension in millimeters. Thereby, the spatial resolution of the acquired video data was determined to be approximately 0.0336 × 0.0336 mm.

Simultaneous data acquisition was repeated for all central structures. Thus, the CAPUT dataset finally consists of six pairs of corresponding FPCT and video data. The data is provided freely available and can be accessed via https://www.github.com/IPMI-ICNS-UKE/CAPUT.

## Experiments: Registration-based Wall Motion Quantification in FPCT data

**Figure 2:**
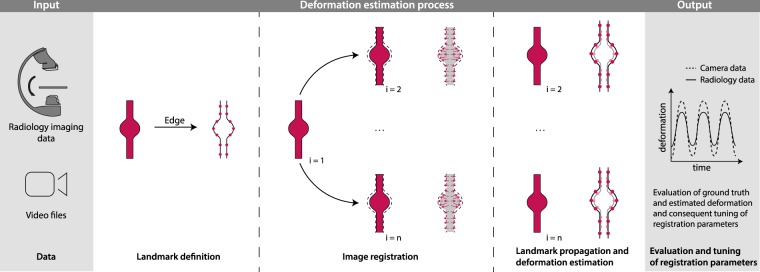
Automatic deformation estimation approach: As input data, radiology imaging data and video files are used. First, using the edge information of the phantom structures, landmarks are automatically generated for image frame *i* = 1. Non-linear registration is then used to compute deformation vector fields with respect to frame *i* = 1 and generated landmarks are then propagated using the computed vector fields. For evaluating the precision of the estimated deformation, the generated video data can be used as ground truth.

During literature review, especially the registration-based wall motion estimation by Oubel *et al*.^12^ appeared promising to us because most parts of their approach were either automated or automatable, rendering the results less observer-dependent than comparable manual thresholding approaches also frequently described in the literature. We, therefore, implemented and slightly extended their registration-based approach for wall motion quantification, and used it as an application example of the benchmark dataset. An overview of the approach is given in Fig. 2. The first step was the definition of pseudo-landmarks on the outer edge of the phantom structures. Therefore, the first frame of minimum expansion during contrast agent inflow was selected as reference image and a Sobel edge filter applied to this image. The resulting edge of the structure of interest was used to sample the sought pseudo-landmarks (approx. 200 randomly chosen edge points; exact number depending on the size of the structure). The second step comprises the main step for estimation of the structure deformation, utilizing non-linear image registration: All frames of the considered 2D + t image sequence are registered to the selected reference image. Due to its excellent performance in different well-known registration challenges^18,19^, the ANTs framework^20^ was applied for registration, using a multi-resolution, symmetric diffeomorphic image registration scheme with mutual information as distance measure. The exact registration parameters are provided in supplemental materials S2. The computed deformation fields of the edge pixels and the sampled pseudo-landmarks finally represent the local wall motion estimation with respect to the selected reference frame.

### Original FPCT resolution experiments

The described registration-based deformation estimation approach was applied to the original FPCT images of the six vessel structures of the CAPUT dataset. To further illustrate the influence of registration parameters on the resulting deformation data, repeated runs were performed with different registration parameters (see supplemental materials S2).

To quantitatively determine the deformation estimation accuracy by employing the high-resolution video data, a two step-procedure was performed. First, maximum structure deformation was assessed using manually placed landmarks. Therefore, a video frame at minimum expansion was selected and around 10–15 landmarks per structure placed on the structure edge. Corresponding landmarks were then manually placed on the structure edge in the next video frame at maximum structure expansion. The average Euclidean distance of the positions of these corresponding landmarks represents a first benchmark of the (maximum) structure deformation. The relatively low number of landmarks could, however, be considered as leading to residual uncertainties of the obtained wall deformation. We, therefore, performed multiple repetitions with different landmark sets, revealing robust and consistent results; respective landmark sets are provided as part of the CAPUT dataset.

To also capture the dynamics of the imaged processes, we further applied the described automated registration-based deformation estimation to the high-resolution video data. Landmark displacements observed for the video data were finally compared to the structure deformation and landmark displacements, respectively, estimated for the FPCT data.

### Experiments with reduced FPCT image resolution

To investigate the feasibility of accurate quantification of geometric deformation of the vessel wall using typical image resolution of different imaging modalities also applied in the given context (e.g. MRI, CT), our high-resolution FPCT imaging data was resampled. In detail, we resampled the FPCT data to an isotropic spatial resolution of 0.37, 0.74 and 1.48 mm/px. Moreover, the original temporal resolution of 25 FPS was reduced to 4 FPS, 2 FPS and 1.5 FPS to cover typical CT, MRI and FPCT literature values. Especially sampling frequencies around 2 FPS are interesting. Given the original dynamic process with an underlying frequency of 1 Hz, it is to be expected that around and especially below this frequency, the reconstruction of the original signal is no longer reliable. This relates to the Nyquist-Shannon sampling theorem^21^, which states that the sampling rate should be larger than twice the highest frequency component of the original signal.

For evaluation of the deformation quantification performance in the presence of reduced FPCT image resolution, we focussed on a structure with comparably high deformation (fusiform aneurysm, feeding vessel inner diameter of 4 mm) and applied the registration parameters that led to minimal errors during the original resolution experiments.

## Results

### Ground truth validation of vessel wall estimation

**Figure 3:**
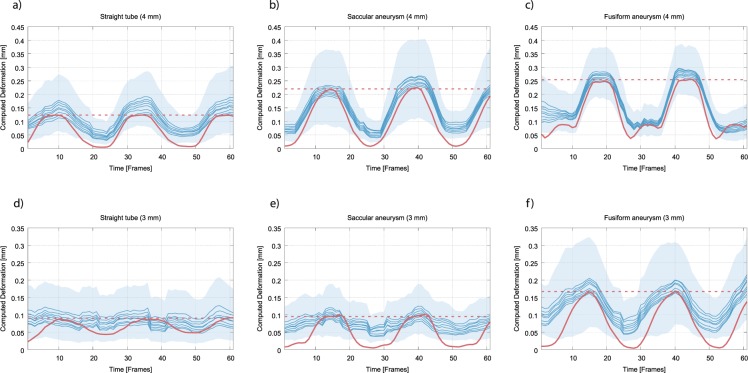
Results of the automatic structure wall deformation estimation. The blue lines represent the results of the registration-based deformation estimation using the FPCT imaging data at original resolution. The dashed red line indicates the mean displacement between minimum and maximum expansion as computed for the landmarks that were manually placed in the video data. The continuous red line further represents the deformation that was determined using the registration-based wall estimation but employing the high-resolution video data. (**a**) Straight tube, inner diameter of 4 mm, (**b**) saccular aneurysm, feeding vessel inner diameter = 4 mm, (**c**) fusiform aneurysm, feeding vessel inner diameter = 4 mm, (**d**) straight tube, inner diameter = 3 mm, (**e**) saccular aneurysm, feeding vessel inner diameter = 3 mm, (**f**) fusiform aneurysm, feeding vessel inner diameter = 3 mm.

The results of the automatic deformation estimation during the inflow of contrast agent for the different structures (artery, saccular aneurysm, fusiform aneurysm) at both inner diameters of the feeding vessel (3 mm, 4 mm) are summarized in Fig. 3. The mean displacement between minimum and maximum structure expansion of the manually placed landmarks is drawn as dashed red line. The continuous red line indicated the registration-based computed average displacement of the pseudo-landmarks with respect to minimum structure expansion as estimated by means of the high-resolution video data. The deformation estimation based on the FPCT imaging data is shown as blue lines (each line indicates the mean landmark displacement with regard to the reference minimum expansion FPCT frame for a specific set of registration parameters; confidence intervals represent the range of the standard deviation of the minimally and maximally estimated deformation). It can be seen that the deformation estimated in the FPCT data is at least in the same order as observed in the ground truth video data for the chosen registration parameters. Especially structure deformation larger than the FPCT spatial resolution data can be accurately reproduced. However, problems arise when the deformation is around or smaller than 0.1 mm; compare, e.g., the results for the straight tube and the saccular aneurysm with feeding vessel inner diameter of 3 mm: While the deformation pattern and its magnitude can still be detected well for the saccular aneurysm, the straight tube deformation as observed by means of the video data cannot be accurately retrieved anymore in the FPCT data. Instead, estimated FPCT deformation values are fluctuating without obvious relation to the continuous red line around a constant level of 0.08 mm, which is more or less the level of the dashed red line. This, however, seems to be a lower boundary of detectable deformation in general (at least for the applied registration approach): Consistently across all structures, obtained mean landmark displacements at the different minimum structure expansion frames average to approximately 0.08 mm (with slight differences, depending on the registration parameters); the expected deformation would be (almost) 0 mm. This, in turn, means that it is plausible that a peak-to-peak structure deformation below this level cannot be reliably estimated.

**Figure 4:**
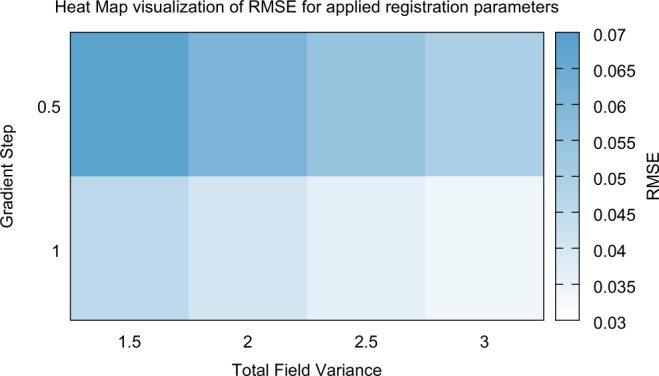
Heat map of the root-mean-square deviation of the ground truth deformation (video data) and the estimated deformation using the FPCT imaging data, evaluated for different registration parameters (gradient step, total field variance).

The spreading of the blue lines, observed for all structures, nevertheless illustrates a dependence of deformation estimation accuracy and registration parameters. Please note that in our case and based on long(er) standing registration experience the registration parameters were already chosen to cover only a relatively small but reasonable range; for inexperienced users, the parameter search space could be far larger. To identify the best registration parameter set (in terms of the most precise wall motion estimation), we calculated the root-mean-square error (RMSE) of the reference deformation data (red continuous line) and parameter-specific results (i. e. the blue lines) for every structure. The RMSE values, averaged across all structures, are plotted in Fig. 4. The horizontal axis of the figure represents the total field variance (i. e. a central regularization parameter), while the gradient step (a main parameter of the optimization approach) is displayed on the vertical axis. It is apparent that a parameter set leading to a minimum error could be successfully identified (gradient step of 1, paired with a total field variance of 3), demonstrating the use of the CAPUT dataset for parameter optimization.

### Performance of registration-based deformation estimation in low(er) resolution imaging data

**Figure 5:**
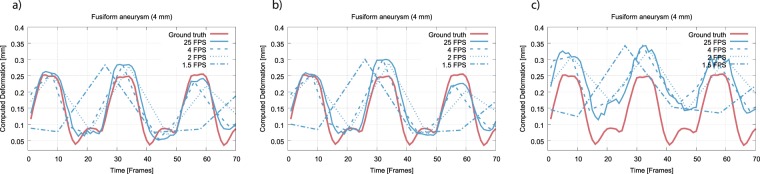
Results of the automatic wall deformation estimation in FPCT data of reduced spatial and temporal image resolution for the fusiform aneurysm data set with 4 mm inner diameter of the feeding vessel. (**a**) Spatial resolution of 0.37 × 0.37 mm; (**b**) 0.74 × 0.74 mm; (**c**) 1.48 × 1.48 mm.

The results of the deformation estimation accuracy evaluation using the downsampled FPCT data are depicted in Fig. 5. Deformation estimation was executed applying the described registration-based approach and using the optimal parameters as determined in the previous section (gradient step: 1, total field variance: 3). Examining the spatially downsampled data at full temporal resolution, the expected effect of a decreasing accuracy with increasing pixel spacing can be observed. The full-resolution video data reveals a maximum structure expansion of around 0.25 mm (red line) with respect to the minimum expansion state. This value can still be approximately extracted using the 0.37 mm/px and 0.74 mm/px resolution. However, at 1.48 mm/px, the maximum deformation magnitude is estimated to be ca. 0.33 mm, which amounts to a 32% overestimation and, hence, a not reliable deformation estimation.

Regarding temporal resampling, the 4 FPS and also in parts the 2 FPS temporal resolution data result in a good fit relative to the full temporal resolution data. Yet, deviations from the ground truth data can still be observed; potential higher frequency motion components (likely due to structure vibration) are, in agreement with the Nyquist theorem, not reflected. Also in agreement with the Nyquist theorem, using the 1.5 FPS dataset, the original signal cannot be reliably retrieved anymore, thereby extensively lowering confidence of the calculated results.

These observations are further supported by the respective RMSE evaluation summarized in Table 1.

**Table 1:** Deviation of the deformation automatically estimated in the full-resolution video data and the deformation estimated in the FPCT data with reduced image resolution, measured as root mean square error.

Spatial resolution	Temporal resolution				
	25 FPS	4 FPS	2 FPS	1.5 FPS	1 FPS
0.37 × 0.37 mm	0.0291	0.0367	0.0603	0.1069	0.1349
0.74 × 0.74 mm	0.0351	0.0410	0.0638	0.1080	0.1334
1.48 × 1.48 mm	0.0884	0.0931	0.1107	0.1175	0.1790

## Discussion

The origin of our work was the wish and demand to develop a deformable flow phantom and measurement setup that allows evaluating computational approaches to assess pulsatile-type cerebral vessel and vessel-like structure wall deformation; the presented data demonstrate successful implementation and data acquisition.

The experimental setup provided us with the means to generate a reliable ground truth and benchmark dataset, enabling us to fine-tune our registration-based deformation estimation. Evaluation of the generated results showed that the estimation of wall motion is possible to a high degree of precision. However, we were also able to demonstrate that the measured deformation (and, hence, deformation estimation accuracy) depends on the chosen registration parameters. This illustrates the need to test approaches for wall motion estimation using respective ground truth data to assess the capability and accuracy limits of the respective approaches. In turn, we hope that other working groups in this field also benefit from the release of the acquired data (consisting of both the FPCT and video data) as a freely accessible benchmark dataset and make use of the CAPUT data. By testing and appropriately tuning of respective deformation estimation approaches using the CAPUT dataset, shortcomings of individual algorithms can be identified and accounted for. In turn, thereby evaluated approaches and parameter settings, i.e. those that are capable of reproducing the geometric movement of the structures in the CAPUT dataset, can be considered to be better suited to reliably and precisely represent wall motion in clinical image data. Therefore, this dataset is expected to be especially interesting for groups working in a clinical setting, since not only wall motion deformation but also derived information such as flow velocity profiles or wall shear stress distributions can be more reliably estimated and interpreted with a higher degree of fidelity by using thoroughly evaluated algorithms and parameter sets. We will further work on complementing the dataset by additional measurements and data and encourage interested readers to contact us in case of specific ideas and desired scenarios.

The second data analysis part addressed the question of the achievable precision of deformation estimation using imaging data with reduced spatial and/or temporal image resolution compared to the CAPUT dataset. Evaluating the full temporal resolution data at different spatial image resolution, even for an image spacing of 1.48 mm/px, a wall motion estimation error up to slightly above 30% was observed. Considering the resolution in relation to the small structure deformation (in the order of 0.25 mm), these results were surprisingly accurate. Therefore, automated assessment of wall motion deformation seems to be feasible even in standard-of-care imaging data when using well-tuned deformation estimation approaches. Yet, it has to be considered that the resulting error is not entirely negligible. Depending on the application (e.g., estimation of wall stresses), the introduced uncertainties and errors will propagate, potentially leading to misleading derived measures that might turn into erroneous conclusions.

The results obtained by analysis of the temporally downsampled data also demonstrated robust performance of the registration-based analysis approach. Although the FPCT framerate was reduced substantially, the maximum deformation could still be retrieved at 4 and 2 FPS with adequate precision. Results at <1.5 FPS revealed substantial difficulties and emphasized the relevance of the Nyquist theorem for the given application. Thus, since most patients have probably a higher resting pulse than 60 bpm (ca. 60–100 bpm), we consider a minimum imaging sampling frequency of 4 FPS to be advisable.

Regarding the deformable structure size, it could be finally argued, that the chosen aneurysm height is relatively small in comparison to aneurysms often observed *in vivo*. Yet, addressed uncertainties of deformation estimation accuracy are especially relevant for smaller structures. It is to be expected that larger aneurysms exhibit stronger deformation, which, in turn, is easier to quantify (see Fig. 3). Moreover, a recent study showed that small (5–9 mm) and very small (<5 mm) aneurysms cause the majority of aneurysmal subarachnoid hemorrhage^22^. Therefore, investigation of small structures is not only very interesting (in the sense of more challenging in terms of associated uncertainties) from an image processing perspective, but also of particular interest from the clinical perspective. Another debatable aspect is the usage of water as a substitute for blood in our experiment since the viscosity of blood is distinctly higher than water. Yet, as our study did not investigate exact flow patterns but only the deformation of the phantom structures, the simplistic usage of water is not expected to have any influence on the presented results.

## Conclusions

In summary, we acquired and provide a benchmark dataset (CAPUT = Cerebral Aneurysm PUlsation Testing) for evaluation and fine-tuning computational approaches for cerebral wall motion estimation and aneurysm deformation, respectively. Our experiments further showed that registration-based deformation estimation using the recorded data is possible to a high degree of precision, even for imaging data with relatively low spatial and/or temporal resolution. However, we also illustrated that parameters for, e.g., registration-based deformation estimation approaches have to be well selected to produce viable results. This, in turn, is precisely the envisioned purpose of the CAPUT dataset, which aims to advance toward reliable and transparent wall motion estimation in standard-of-care images and the development of respective standardized methods that potentially allow incorporating wall deformation measures into treatment decision workflows.

## Electronic supplementary material

Appendix

Examplary video file of a flow phantom structure
