# Supplementary material for: A new cerebral vessel benchmark dataset (CAPUT) for validation of image-based aneurysm deformation estimation algorithms
Source: Sci Rep. 2018 Oct 30;8:15999. doi: 10.1038/s41598-018-34489-2 (PMC6207668; doi:10.1038/s41598-018-34489-2)
Supplement: Supplementary file 1 — Appendix [file 41598_2018_34489_MOESM1_ESM.pdf]

A new cerebral vessel benchmark dataset (CAPUT) for validation of image-based aneurysm deformation estimation algorithms

Daniel Schetelig[1,*], Andreas Frölich[2], Tobias Knopp[3,4], and René Werner[1]

[1]University Medical Center Hamburg-Eppendorf, Department of Computational Neuroscience, Hamburg, 20246, Germany
[2]University Medical Center Hamburg-Eppendorf, Department of Diagnostic and Interventional Neuroradiology, Hamburg, 20246, Germany
[3]University Medical Center Hamburg-Eppendorf, Section for Biomedical Imaging, Hamburg, 20246, Germany
[4]Hamburg University of Technology, Institute for Biomedical Imaging, Hamburg, 20246, Germany
[*]d.schetelig@uke.de

Supplemental material S1

Analytical estimation of the expected deformation is conducted using the example of a fusiform aneurysm, since its symmetry alleviates analytical consideration. The symmetry of the sphere leads to the assumption that the tangential stress σ_t is uniform (see Fig. 1a).

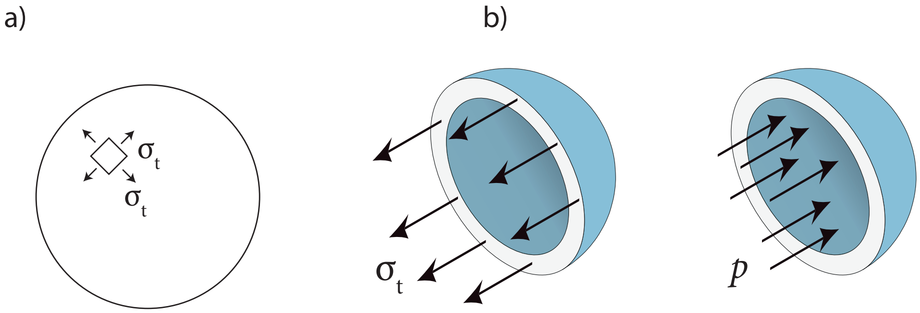

Figure 1. Free body diagrams. (a) Thin-walled sphere under inner pressure, (b) Free body diagram of the sphere under pressure.

The free body diagram of the sphere (see Fig. 1b) leads to the following equilibrium:

$$p \cdot \frac{\pi d_i^2}{4} = \sigma_t \frac{\pi}{4} \cdot \left(d_a^2 - d_i^2\right) \tag{1}$$

$$= \sigma_t \cdot \frac{\pi}{4} \cdot \left(d_i^2 + 4 \cdot d_i \cdot s + 4 \cdot s^2 - d_i^2\right), \tag{2}$$

with d_i: inner diameter, d_a: outer diameter, p: internal pressure and σ_t: tangential stress.

Since the structure is thin walled, s^2 can be neglected, leading to

$$\sigma_t = p \cdot \frac{d_i}{4 \cdot s}. \tag{3}$$

Assuming a pressure of $p = 0.025\ \frac{N}{\mathrm{mm}^2}$ (considering the maximum pressure of the pump and decreases in pressure due to friction), a wall thickness of $s = 1$ mm and a inner diameter of $d_i = 11$ mm, this computes a stress of $\sigma_t = 0.06875\ \frac{N}{\mathrm{mm}^2}$. Using Hooke's law (with a Young's modulus of $E = 3\ \frac{N}{mm^2}$), this equates to a deformation of $\Delta l = 0.252$ mm.

Supplemental material S2

Registration call:

```
Ants_Reg_Call="${Executable} --dimensionality 2 \
--output [$OutputDirectory/fixedImage_to_${i}_] \
--metric MI[$fixedImg,$movingImg,1,32,Regular,0.25] \
--transform SyN[$gradientStep,$updateFieldVariance,
    $totalFieldVariance] \
--convergence [250x250x250x250,1e-6,10] \
--shrink-factors 8x4x2x1 \
--smoothing-sigmas 3x2x1x0vox"
```

gradient step: 0.5 or 1.0
update field variance: 0
total field variance: varied between 1.5 and 3.0.
